# Can the ultrasound microcystic pattern accurately predict borderline ovarian tumors?

**DOI:** 10.1186/s13048-023-01253-8

**Published:** 2023-08-11

**Authors:** Danyi Liu, Guorong Lyu, Hongwei Lai, Liya Li, Yaduan Gan, Shuping Yang

**Affiliations:** 1 Department of Ultrasound, The Second Affiliated Hospital of Fujian Medical University, Quanzhou, Fujian China; 2 Department of Ultrasound, Collaborative Innovation Center for Maternal and Infant Health Service Application Technology, Quanzhou Medical College, No.2, Anji Road, Quanzhou, Fujian China; 3 Department of Ultrasound, Fujian Provincial Maternity and Children’s Hospital, Fuzhou, Fujian China; 4 Department of Ultrasound, Zhangzhou Affiliated Hospital of Fujian Medical University, Zhangzhou, Fujian China

**Keywords:** Borderline ovarian tumors, Ultrasound, Microcystic pattern, Pathology

## Abstract

**Objective:**

To investigate whether the ultrasound microcystic pattern (MCP) can accurately predict borderline ovarian tumors (BOTs).

**Methods:**

A retrospective collection of 393 patients who met the inclusion criteria was used as the study population. Indicators that could well identify BOT in different pathological types of tumors were derived by multivariate unordered logistic regression analysis. Finally, the correlation between ultrasound MCP and pathological features was analyzed.

**Results:**

(1) MCP was present in 55 of 393 ovarian tumors, including 34 BOTs (34/68, 50.0%), 16 malignant tumors (16/88, 18.2%), and 5 benign tumors (5/237, 2.1%). (2) Univariate screening showed significant differences (*P* < 0.05) in patient age, CA-125 level, ascites, > 10 cyst locules, a solid component, blood flow, and MCP among BOTs, benign ovarian tumors, and malignant ovarian tumors. (3) Multivariate unordered logistic regression analysis showed that the blood flow, > 10 cyst locules, and MCP were significant factors in identifying BOTs (*P* < 0.05). (4) The pathology of ovarian tumors with MCP showed "bubble"- or "fork"- like loose tissue structures.

**Conclusion:**

MCP can be observed in different pathological types of ovarian tumors and can be used as a novel sonographic marker to differentiate between BOTs, benign tumors and malignant tumors. MCP may arise as a result of anechoic cystic fluid filling the loose tissue gap.

## Introduction

Borderline ovarian tumors (BOTs) were first proposed in 1929 [1]. An intermediate tumor type between benign and malignant, BOTs exhibit cytologic features indicative of malignancy without destructive mesenchymal infiltration and have a slow clinical progression [2]. Among all BOTs, serous and mucinous tumors are the most common, while other pathological types are rare [3]. Due to the significant differences in pathological features and clinical manifestations, different histological types should be evaluated separately during the assessment of BOTs [4]. Serous BOTs often present as unilocular or multilocular solid cysts with more papillae and more blood flow signals within the papillae, while mucinous BOTs tend to have more septa and cyst locules [5–7].

The International Ovarian Tumor Analysis (IOTA) proposed the Assessment of Different NEoplasias in the adneXa (ADNEX) model in 2014, which can easily identify benign and malignant ovarian tumors but has difficulty in correctly differentiating between BOTs and stage I primary invasive malignant tumors [8–10]. Recently, Timor-Tritsch et al. [11] identified the microcystic pattern (MCP) and found that it was not present in a random sample of 20 cases of both benign cystadenomas and ovarian epithelial carcinomas. Although this feature shows excellent specificity for BOTs, the generalizability of the findings remains to be externally validated. Our team analyzed and compared the ultrasound characteristics of benign epithelial tumors, primary malignant epithelial tumors, and BOTs, and the results showed that the presence of MCP was an independent risk predictor for BOTs [12]. Therefore, in accordance with the consensus of the IOTA group on adnexal tumors [13], this study aimed to explore whether MCP and other ultrasound features are helpful in the diagnosis of BOTs among a larger range of pathological types and to investigate whether MCP is a specific ultrasound sign of BOTs by analyzing the proportion of cases with MCP in benign ovarian tumors, BOTs, and malignant ovarian tumors. In addition, this study explored the association between MCP ultrasound features and pathological image features.

## Materials and methods

### Patients and data acquisition

This retrospective study was approved by the Institutional Review Boards (IRBs) of the Second Affiliated Hospital of Fujian Medical University (IRB No. 2022519), and the requirement for written informed consent was waived. Patients with ovarian tumors who were diagnosed at our hospital and the hospital of Zhangzhou City, Fujian Medical University, from January 2016 to June 2022 and confirmed by surgical pathology results were recruited. The inclusion criterion was the presence of a mass in the adnexal region on ultrasound images. When multiple lesions were present in the adnexal region, the lesion with the most complex ultrasound presentation or the one with the largest mass if the ultrasound presentations were similar was selected. The exclusion criteria were as follows: (1) patients who did not undergo ultrasound examination at our hospital within 3 months prior to the operation; (2) patients whit incomplete ultrasound images and reports or those from which needed ultrasound features could not be extracted; (3) patients with unclear postoperative pathology reports; and (4) patients with pathological findings of one lesion showing the presence of two or more pathological types at the same time.

Clinical data, ultrasound data, and pathology data of patients were extracted from inpatient or outpatient records. A total of 393 cases were finally included in the study, including 237 cases of benign tumors, 68 cases of BOTs, and 88 cases of malignant tumors.

### Ultrasound data collection

Ultrasound equipment included GE-Voluson E10, Mindray Resona I9, and other color Doppler ultrasound diagnostic devices with abdominal probe frequencies of 3.5–6.5 MHz and intracavitary probe frequencies of 5–9 MHz. We retrieved ultrasound images of patients who met the inclusion criteria and recorded the ultrasound features.

Ascites was defined as fluid in locations other than the rectal trap of the uterus [14]. A solid component was defined as the presence of papillary projections on the cyst wall or other solid components within the mass [13]. Papillary projections were defined as solid components of the cyst wall convex to the cystic lumen ≥ 3mm [13]. Septum thickness ≥ 3mm was considered a thick septum [15]. The color score (CS) was defined as follows: CS = 1 for no color Doppler flow within the entire lesion (wall and/or internal component); CS = 2 for minimal flow; CS = 3 for moderate flow; and CS = 4 for very strong flow [12]. In this study, the blood flow signals were grouped according to two different methods: (1) CS = 1 in the no blood flow group and CS = 2, 3 and 4 in the blood flow group, and (2) CS = 1 and 2 in the sparse blood flow group and CS = 3 and 4 in the abundant blood flow group. Cysts were considered irregular if they had papillae on the inner wall, and solid tumors or solid components were considered irregular if they had irregular contours [13]. Acoustic shadowing was defined as echogenic loss behind the mass [14]. MCP was defined as a thin-walled microcyst-like structure of 1–3 mm appearing on the solid component of the tumor, papillary projection, or cyst wall/septum [11]. The IOTA staging of the tumors was strictly classified as specified in the IOTA panel consensus on ovarian tumors.

Two sonographers with more than 5 years of experience in gynecologic ultrasound analyzed the tumor for MCP and other ultrasound features, and the final results were further confirmed by a specialist with more than 15 years of experience in gynecologic ultrasound. The three analysts were ignorant of the pathological results of the tumor. The postoperative pathological findings were used as the gold standard and the ultrasound features were compared against the pathological images.

### Statistical methods

SPSS 26.0 was used for statistical processing. Continuous variables with normal distributions are expressed as the means and standard deviations, continuous variables with nonnormal distributions are expressed as medians and quartiles, and categorical variables are expressed as frequencies and percentages. Nonparametric tests were used for continuous variables, and chi-square tests or Fisher's exact probability method were used for categorical variables. Variables with *P* < 0.05 for two-way comparisons among the three groups were selected for inclusion in the multivariate unordered logistic regression analysis. *P* < 0.05 was considered statistically significant.

## Results

A total of 393 cases of ovarian tumors were included in this study, and the clinical baseline information of the patients is shown in Table 1. Patient age and serum CA-125 levels were significantly different among patients with benign tumors, BOTs, and malignant tumors (*P* < 0.05). In contrast, the proportion of postmenopausal patients was not significantly different between patients with borderline and malignant tumors (*P* > 0.05).

**Table 1:** Clinical base information of patients with different pathological types of tumors

	Benign (*n* = 237)	Borderline (*n* = 68)	Malignant (*n* = 88)
Age (years)	37.6 ± 11.9	44.4 ± 15.5^a^	50.8 ± 12.4^a,b^
Pre/post-menopausal (cases)	188/49	40/28^a^	41/47^a^
CA-125 (U/ml)	22.4(14.1 ~ 43.8)	32.0(15.0 ~ 89.1)^a^	96.0(25.0 ~ 244.0)^a,b^

^a^*P*<0.05 compared with benign tumors

^b^*P*<0.05 compared with BOTs

The IOTA staging of different pathological types of tumors is shown in Table 2. According to this staging method, benign ovarian tumors tended to present as unilocular masses (uniocular cyst (35.0%), uniocular-solid cyst (23.2%), solid tumor (7.6%)). BOTs mostly presented as cystic solid masses (uniocular-solid cyst (44.1%), multilocular-solid cyst (39.7%), and solid tumor (0%)). The proportion of solid masses was more prominent in malignant ovarian tumors (solid tumor (22.7%)) than in the other tumor types. There were significant differences between benign ovarian tumors, BOTs and malignant ovarian tumors in IOTA staging (*P* < 0.05).

**Table 2:** IOTA staging of different pathological types of tumors

IOTA staging	Pathology results		
	Benign (*n* = 237)	Borderline (*n* = 68)^a^	Malignant (*n* = 88)^a,b^
Unilocular cyst	83(35.0%)	2(2.9%)	0(0%)
Unilocular-solid cyst	55(23.2%)	30(44.1%)	36(40.9%)
Multilocular cyst	41(17.3%)	9(13.2%)	2(2.3%)
Multilocular-solid cyst	40(16.9%)	27(39.7%)	30(34.1%)
Solid tumor	18(7.6%)	0(0%)	20(22.7%)

^a^*P*<0.05 compared with benign tumors

^b^*P*<0.05 compared with BOTs

The differences in ultrasound features between benign ovarian tumors, BOTs and malignant ovarian tumors according to the morphological indices described in the consensus of the IOTA group on ovarian tumors are shown in Table 3. Ascites, > 10 cyst locules, presence or absence of solid components and blood flow, and MCP differed significantly in different pathological tumor types (*P* < 0.05). In particular, > 10 cyst locules and MCP were found more often in BOTs, and ascites, solid components, and blood flow signals were found more often in malignant ovarian tumors.

**Table 3:** Clinical base information of patients with different pathological types of tumors

	Pathology results		
	Benign (*n* = 237)	Borderline (*n* = 68)	Malignant (*n* = 88)
With/without ascites (cases)	16/221	12/56^a^	28/60^a,b^
Maximum diameter of lesion (mm)	70.0(50.6 ~ 98.8)	121.9(77.3 ~ 180.6)^a^	119.0(81.4 ~ 152.0)^a^
Absence/presence > 10 cyst locules (cases)	231/6	52/16^a^	80/8^a,b^
With/without solid components (cases)	115/122	60/8^a^	85/3^a,b^
Diameter of largest solid component (mm)	29.6(18.1 ~ 46.9)	33.5(14.0 ~ 67.0)	558.0(38.4 ~ 85.5)^a,b^
With/without papillary projection (cases)	64/173	52/16^a^	59/29^a^
Yes/No ≥ 3 papillary projections (cases)	33/204	15/53	30/58^a^
Yes/No thick septum exists(cases)	58/179	17/51	39/49^a,b^
With/without blood flow (cases)	29/208	31/37^a^	66/22^a,b^
Sparse/abundant blood flow (cases)	232/5	62/6^a^	72/16^a^
Tumor regular/irregular (cases)	151/86	12/56^a^	9/79^a^
With/without acoustic shadows (cases)	41/196	7/61	14/74
With/without MCP (cases)	5/232	34/34^a^	16/72^a,b^

^a^*P*<0.05 compared with benign tumors

^b^*P*<0.05 compared with BOTs

The independent variables of patient age, serum CA-125 level, ascites, > 10 cyst locules, presence of solid components, blood flow, and MCP were included in the multivariate unordered logistic regression analysis, and the results of their likelihood ratio tests are shown in Table 4. The results showed that the *P* value of the likelihood ratio test was less than 0.05 for all independent variables, except for ascites, indicating that these independent variables had a significant effect on the regression results.

**Table 4:** Assignment of independent variables and likelihood ratio test results

Independent variable	Assignment	Likelihood ratio test	
		χ^2^	*P*
Age		29.085	< 0.001
CA-125		11.046	0.004
Present ascites	"Yes" = 1,"No" = 2	2.248	0.325
Present > 10 cyst locules	"Yes" = 1,"No" = 2	11.858	0.003
Present solid components	"Yes" = 1,"No" = 2	25.441	< 0.001
Present flow	"Yes" = 1,"No" = 2	36.884	< 0.001
Present MCP	"Yes" = 1,"No" = 2	28.825	< 0.001

The logistic regression results for each independent variable are shown in Tables 5 and 6. The presence of blood flow, > 10 cyst locules and MCP can be used as independent risk predictors for BOTs (*P* < 0.05). When benign ovarian tumors were used as the reference group, all of the above features were risk factors. When ovarian malignancy was used as the reference group, > 10 cyst locules and MCP were risk factors, and the presence of blood flow was a protective factor. MCP can be an independent risk predictor of BOTs compared with benign tumors and malignant tumors (*P* < 0.05).

**Table 5:** Significant indicators and OR among BOTs compared to benign tumors

	B	Wald χ^2^	*P*	OR	95%CI
Age	0.050	11.624	0.001	1.051	1.021 ~ 1.081
CA-125	0.003	6.565	0.010	1.003	1.001 ~ 1.005
Present > 10 cyst locules	2.159	10.742	0.001	8.662	2.382 ~ 31.500
Present solid components	0.899	2.952	0.086	2.457	0.881 ~ 6.851
Present flow	0.973	4.732	0.030	2.647	1.101 ~ 6.361
Present MCP	2.313	22.621	< 0.001	10.106	3.896 ~ 26.217

Benign tumors were used as the reference group

**Table 6:** Significant indicators and OR among BOTs compared to malignant tumors

	B	Wald χ^2^	*P*	OR	95%CI
Age	-0.018	1.235	0.266	0.982	0.952 ~ 1.014
CA-125	0.000	0.656	0.418	1.000	0.977 ~ 1.000
Present > 10 cyst locules	1.218	4.050	0.044	3.381	1.032 ~ 11.077
Present solid components	-1.773	5.425	0.020	0.170	0.038 ~ 0.755
Present flow	-1.483	10.293	0.001	0.227	0.092 ~ 0.562
Present MCP	1.967	16.195	< 0.001	7.146	2.742 ~ 18.621

Malignant tumors were used as the reference group

Of the 393 patients included in the study, a total of 55 patients had MCP, including 34 (34/68, 50.0%) BOTs (21 serous BOTs, 11 mucinous BOTs, 1 seromucinous BOT, and 1 clear cell borderline tumor), 16 (16/88, 18.2%) malignant tumors (5 serous carcinomas, 2 mucinous carcinomas, 4 clear cell carcinomas, 3 endometrioid carcinomas, 1 metastasis to ovary, and 1 granulosa cell tumor) and 5 (5/237, 2.1%) benign tumors (1 mature teratoma, 1 mucinous cystadenoma, 1 struma ovarii, 1 corpus luteum cyst, and 1 sclerosing stromal tumor). The proportion of MCP in serous BOTs was higher than that in mucinous BOTs (*P* < 0.05). The specific pathological types are shown in Table 7.

**Table 7:** Different pathological types and MCP

Type of pathology	With MCP	Without MCP	Total
BOTs			
Serous borderline tumor	21	13	34
Mucinous borderline tumor	11	19	30
Endometrioid borderline tumor	0	2	2
Seromucinous borderline tumor	1	0	1
Clear cell borderline tumor	1	0	1
Benign tumor			
Mature teratoma	1	71	72
Endometrial cyst	0	77	77
Serous cystadenoma	0	24	24
Mucinous cystadenoma	1	18	19
Struma ovarii	1	0	1
Fibromatosis	0	2	2
Ovarian cyst	0	10	10
Follicle cyst	0	8	8
Corpus luteum cyst	1	14	15
Tubovarian abscess	0	1	1
Seromucinous cystadenoma	0	2	2
Serous adenofibroma	0	4	4
Sclerosing stromal tumor	1	1	2
Malignant tumor			
Serous carcinoma	5	32	37
Mucinous carcinoma	2	9	11
Immature teratoma	0	7	7
metastasis to ovary	1	10	11
Endometrioid carcinoma	3	2	5
Clear cell carcinoma	4	7	11
Granulosa cell tumor	1	3	4
Dysgerminoma	0	1	1
Malignant Brenner tumor	0	1	1
Total	55	338	393

**Fig. 1:**
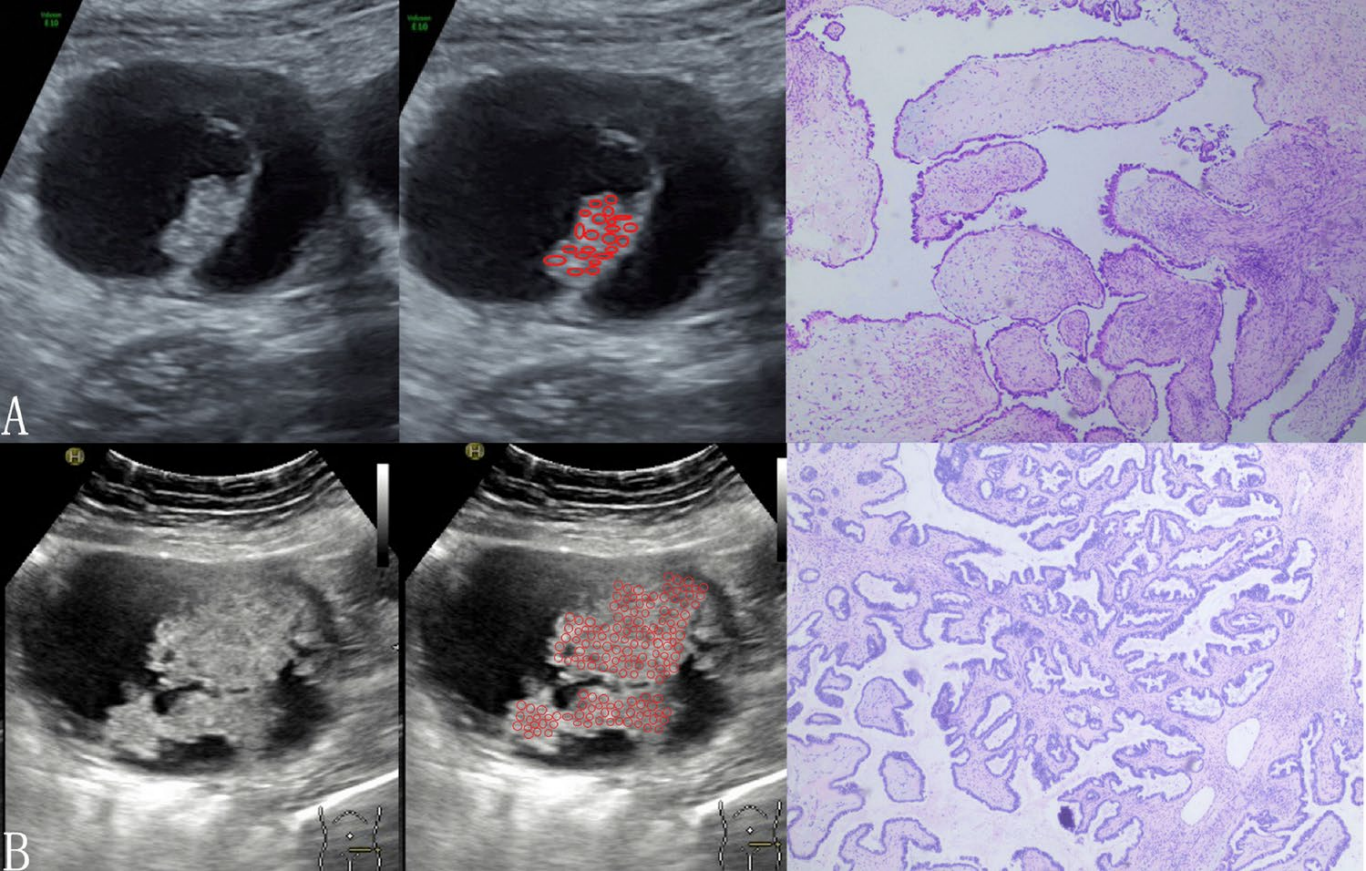
Ultrasound images of ovarian tumors with MCP with pathological controls. The microvesicles in MCP are marked with red circles. On the pathological image, some of the tumors appear as "bubble"-like structures, such as soap bubbles stacked together, with large gaps between the "bubbles" (**A**). Some of the tumors appear pathologically like a "fork of a tree", with the papillae exhibiting multilevel branching, thin papillae and dense branching, but the tissue gap is still large (**B**). **A** Serous borderline tumor; **B** serous borderline tumor

**Fig. 2:**
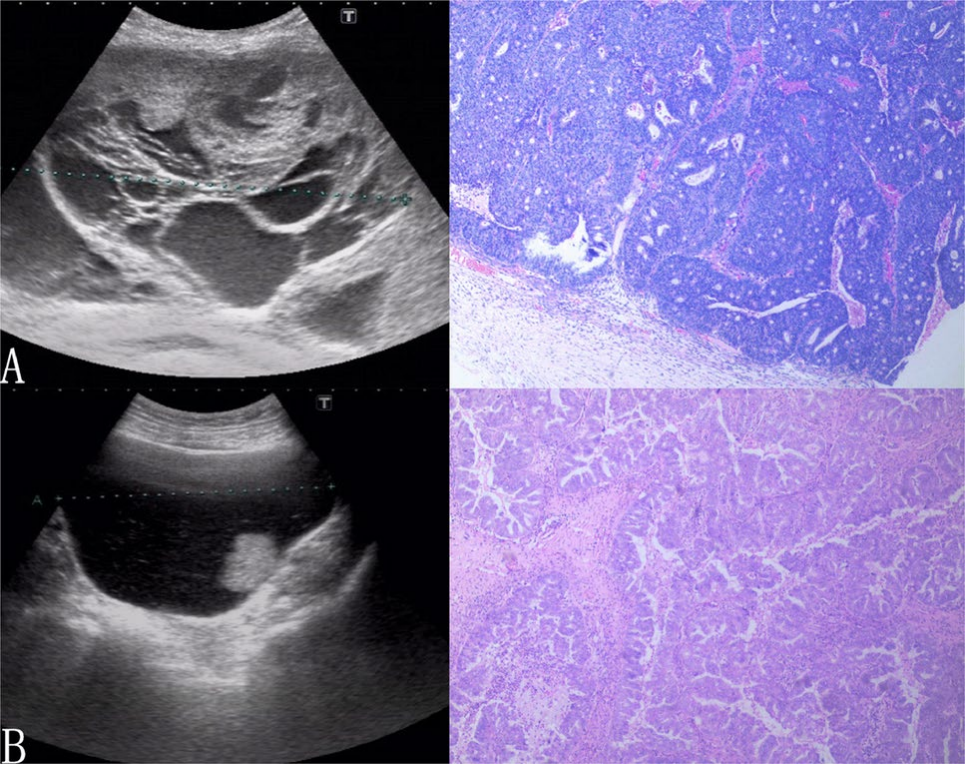
Ultrasound images of ovarian tumors without MCP with pathological controls. The pathology of ovarian tumors without MCP shows a denser histological structure with smaller intertissue spaces. **A** High-grade serous carcinoma; **B** high-grade serous carcinoma

The pathological images of ovarian tumors with MCP (55 cases) were analyzed, and it was found that 21 cases showed a "bubble"-like structure under low magnification, resembling soap bubbles stacked together with large gaps between the "bubbles" (Fig. 1A). In 34 cases, the tumors were similar to "tree branches", and the papillae showed multilevel branching, with thin papillae and dense branches, but the tissue gap was still large (Fig. 1B). In contrast, the pathological images without MCP features (338 cases) were more densely organized with smaller intertissue spaces (Fig. 2). Notably, four of the serous BOTs showed "bubble-like" structures at low magnification but no MCP on ultrasound images.

## Discussion

BOTs occur more frequently in young women, are often associated with infertility, have a high rate of early diagnosis, and have a better prognosis. Many patients with BOTs often opt for fertility-preserving procedures [16]. Therefore, the preoperative diagnosis of BOTs is particularly important. Some scholars believe that the most common ultrasound features of BOTs are papillary protrusions within cysts with internal blood flow signals; however, this feature is also found in some benign epithelial tumors and malignant ovarian tumors and cannot be used as a specific diagnostic marker for BOTs [17–19]. Recently, it has been proposed that MCP within the tumor is a characteristic ultrasound manifestation of BOTs, and this new feature can help to correctly identify BOTs and distinguish them from ovarian cancer and benign ovarian lesions, but the results remain to be validated.

In this study, we retrospectively collected ovarian tumor cases to determine whether MCP can be used as a specific ultrasound indicator for BOTs in a wider range of pathological types. The results showed that patient age, CA-125 level, > 10 cystic chambers, presence of blood flow, and MCP could properly differentiate BOTs from benign tumors (*P* < 0.05). The presence of a solid component, blood flow, MCP, and > 10 compartments discriminated BOTs from malignant tumors (*P* < 0.05). In the logistic regression model LR2 proposed by IOTA [20] and the ADNEX model [21], patient age, CA-125 level, and > 10 cyst locules were included as predictors, and the results of the present study support these findings. According to the simple rules (SR) for ovarian tumor classification [22] and the O-RADS scoring system [23], the present study classified CS = 1 and CS = 2, 3 and 4 into groups with or without blood flow and CS = 1 and 2 and CS = 3 and 4 into groups with sparse or abundant blood flow, respectively. The comparison revealed that the presence or absence of a blood flow signal was significantly different in the three pathological types of tumors (*P* < 0.05), while sparse or abundant blood flow did not distinguish well between borderline and malignant tumors (*P* > 0.05), which was also slightly different from our team's previous findings [12].

MCP was observed in different pathological types of tumors, but there were differences in its proportion, and even in a larger range of case types, MCP can still be considered an independent risk predictor for BOTs. This is different from the results of Timor-Tritsch et al. [11]. Of their 20 randomly selected cases of ovarian cystadenoma with ovarian epithelial cell carcinoma, none of them had MCP. The reason for this discrepancy may be due to the small number of benign versus malignant tumors included in their study and the fact that the type of pathology was limited to ovarian epithelial tumors. Past studies by our team [12] showed that the presence of MCP was higher in BOTs than in benign epithelial tumors versus malignant epithelial tumors. However, in this study, when the pathological type was not limited to epithelial tumors, MCP was also observed in some ovarian granulosa cell tumors, mature teratomas, and other types of tumors. This may be due to the wide variety of ovarian tumors and a certain heterogeneity in the presentation of different pathological types of tumors.

A study by Landolfo et al. [24] found the presence of small echogenic areas within the papillae of unilocular-solid cysts, which correlated with the malignancy of the tumors. Small echogenic areas are most commonly seen in papillary protuberances of BOTs (63%), but are not uncommon in malignant tumors (58%), and because the study did not clearly define small anechoic areas, it was not possible to determine whether they were different from the MCP described in this study. Virgilio et al. [25] also observed tiny vesicular anechoic areas in the papillary projections of cystic adenofibromas and concluded that the pathological basis of this feature is internal papillary edema.

The results of this study showed that the pathology of ovarian tumors with MCP showed a "bubble"- or "fork"- like loose tissue structure with more large gaps. In general, the cystic fluid appears anechoic under ultrasound, and these cysts fill the interstitial space, perhaps forming the basis for MCP. This is similar to the "soap bubble"-like stacking pathology observed by Timor-Tritsch et al. [11], but they suggest that it is these "soap bubbles" that form the MCP. It is worth mentioning that four cases of serous BOTs showed "bubble"-like structures under low magnification, but the ultrasound images did not show MCP, perhaps due to different ultrasound instruments, examination surfaces or lesion depths or lower probe frequencies. The pathological basis of MCP remains to be further explored.

In addition, an interesting result was observed in this study, where the percentage of MCP was higher in serous BOTs than in mucinous BOTs (*P* < 0.05), which may imply that MCP is a specific ultrasound manifestation of serous BOTs, the confirmation of which may require additional external validation. This result may be based on the pathological characteristics of serous BOTs with layered branching of papillae and pseudopapillae resulting in a lax histological architecture.

The current study also had some limitations. As it was a retrospective study, many past ultrasound images did not have the complete information we needed or were excluded due to the difficulty in obtaining complete clinical cases; thus, the sample size was small. Of all the malignant ovarian tumors we included, 29 cases (29/88,33.0%) were advanced malignant tumors. However, the diagnosis of advanced malignancy was not difficult, and we did not perform a targeted study of stage I and II ovarian cancer, so we cannot conclude whether the microcystic sign can help to distinguish BOTs from early ovarian tumors. Furthermore, the MCPs observed in this study all showed a thin-walled microcyst-like structure of 1–3 mm appearing on the solid component of the tumor, papillary projection, and cyst wall/septum, and the presence of a single MCP was not observed, probably due to the small sample size and insufficient frequency of the probe. These conditions need to be further verified by subsequent multicenter prospective clinical trials.

## Conclusion

MCP can be observed in BOTs and benign and malignant tumors, but it can still be a novel sonographic marker for BOTs even among a wider range of pathological types (*P* < 0.05). In addition, > 10 cyst locules and the presence of blood flow can be used as independent risk predictors for BOTs (*P* < 0.05). The pathological basis of MCP may be due to the anechoic cystic fluid filling the lax tissue gap.

## Data Availability

The datasets used and/or analysed during the current study are available from the corresponding author on reasonable request.

## Abbreviations

MCP: Microcystic pattern
BOTs: Borderline ovarian tumors
IOTA: International Ovarian Tumor Analysis
ANDEX: Assessment of Different NEoplasias in the adneXa
IRBs: Institutional Review Boards
